# A Mistaken Diagnosis of Secondary Glioblastoma as Parasitosis

**DOI:** 10.3389/fneur.2019.00952

**Published:** 2019-09-06

**Authors:** Chenxi Liu, Wenlong Xu, Pan Liu, Yukui Wei

**Affiliations:** Department of Neurosurgery, Xuanwu Hospital, Capital Medical University, Beijing, China

**Keywords:** secondary glioblastoma, brain neoplasm, parasitosis, mistaken diagnosis, multi-parametric MRI, FDG-PET, FET-PET

## Abstract

**Background:** Glioblastoma is a malignant brain tumor with poor prognosis requiring early diagnosis. Secondary glioblastoma refers to cases that progressed from low-grade glioma. Evidence shows that timely resection correlates with increased survival.

**Case presentation:** We describe a case of a patient with secondary glioblastoma who was mistakenly diagnosed with *Angiostrongylus cantonensis* infection until 7 years after disease onset. The patient presented with non-specific clinical manifestations at disease onset. A conventional magnetic resonance imaging (MRI) in the primary survey provided insufficient information, and thus failed to identify the malignancy. During follow-up, unfortunately, clinicians were misled by the patient's raw food diet, a positive serum parasite antibody and a result of low glucose metabolism on Fluorodeoxyglucose-positron emission tomography-computed tomography (FDG-PET-CT). The patient was diagnosed with parasitosis. However, his condition kept getting worse under antiparasitic treatment. Preoperative magnetic resonance spectroscopy (MRS) and diffusion tensor imaging (DTI) failed to reverse the mistaken impression. Final diagnosis was confirmed until intraoperative and postoperative pathological findings indicated glioblastoma.

**Conclusion:** We ascribe the incorrect diagnosis to insufficient understanding on imaging manifestations of brain neoplasm as well as clinical features of parasitosis. Thus, we review the MRI, FDG-PET-CT, MRS, and DTI data of this case according to the timeline, refer to relevant studies, and point out the pitfalls. With a long course of slowly progressing, this was a rare case of secondary glioblastoma with the absence of isocitrate dehydrogenase 1 (IDH1) gene mutation.

## Introduction

Glioblastoma is the most common type of malignant brain tumor. The overall survival of patients with glioblastoma is extremely poor, with a survival rate of 42.4% at 6 months, 17.7% at 1 year, and 3.3% at 2 years (1). Secondary glioblastoma refers to cases that have progressed slowly from low-grade astrocytoma. According to the SEOM clinical guidelines of low-grade gliomas (LGGs) (2), surgical resection is the first step when dealing with LGGs, and the overall patient survival is increased after a thorough resection. Thus, early diagnosis of gliomas is critical. Here, we report a case of secondary glioblastoma that was mistakenly diagnosed with *Angiostrongylus cantonensis* infection, which affected our ability to properly treat the patient.

## Case Report

A 53-year-old male from southwestern China was admitted to our hospital on September 21, 2018 with a complaint of intermittent headaches for 7 years and right leg weakness for 6 months. The patient often consumed sashimi, raw oysters, bullfrogs, and snakes, and before the onset of the symptoms, he consumed raw beef. The patient had a long history of seeking medical help without significant alleviation of the major symptoms.

**Figure 1:**
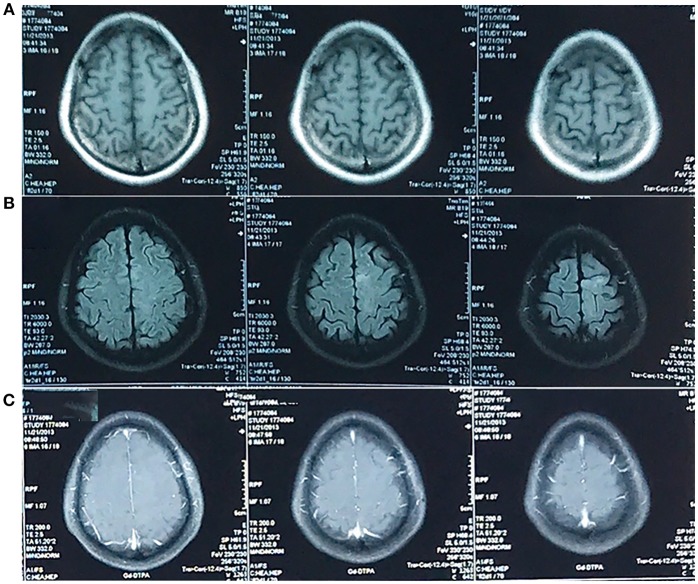
MRI in November 2013. An abnormal signal was confined to left frontal lobe. It was not obvious on the T1-weighted image **(A)** and hyperintense on the T2-weighted-Fluid-Attenuated Inversion Recovery (T2-FLAIR) image **(B)** in the absence of enhancement **(C)**. The T2-weighted image was unavailable.

In March 2011, the patient presented with an intermittent headache that was largely confined to the cranial vertex. In November 2013, he underwent a magnetic resonance imaging (MRI) scan that was unremarkable, except for the presence of small patchy areas in the left frontal lobe (Figure 1). The patient was prescribed symptomatic drugs and his condition improved.

**Figure 2:**
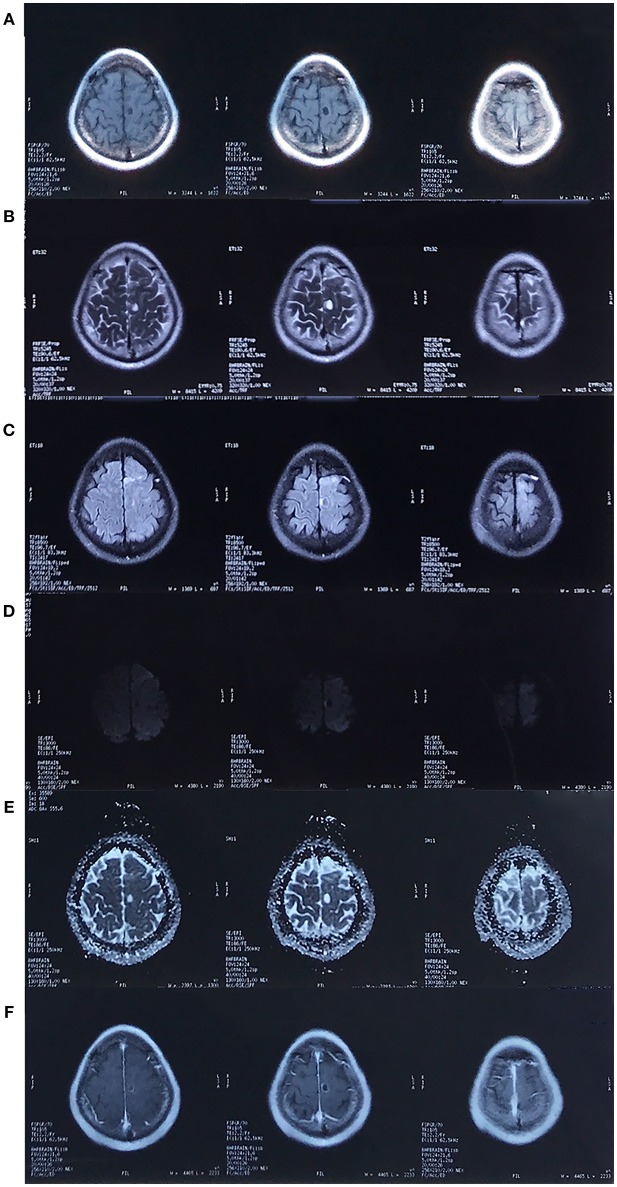
MRI in October 2017. There was a cystic lesion in the left frontal lobe that presented as hypointense on the T1-weighted image **(A)**, hyperintense on the T2/T2-FLAIR/Apparent diffusion coefficient (ADC) image **(B,C,E)** and hypointense on the diffusion weighted imaging (DWI, **D**), with surrounding edema and rim enhancement **(F)**.

In October 2017, however, his headache returned, and the pain was more severe. He underwent another MRI scan, revealing a cystic lesion with rim enhancement at the same location (Figure 2).

**Figure 3:**
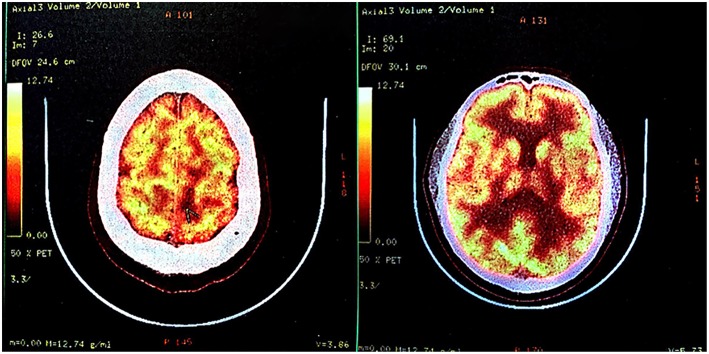
Fluorodeoxyglucose-positron emission tomography-computed tomography (FDG-PET-CT) in October 2017. FDG-PET-CT indicated low glucose metabolism of left frontal-parietal lobe, which was suggestive of an intracranial primary benign lesion.

Fluorodeoxyglucose-positron emission tomography-computed tomography (FDG-PET-CT) showed low metabolism (Figure 3), and coupled with the patient's raw food diet, a diagnosis of parasitosis was made. The patient was positive for *A. cantonensis* antibodies in the serum, but not in the cerebrospinal fluid. However, DNA test for *A. cantonensis* by polymerase chain reaction (PCR) was negative. The patient was prescribed albendazole, a broad-spectrum anthelmintic, and his condition improved after five courses. Unfortunately, he began to complain of right leg weakness. An MRI scan performed in March 2018 showed an enlarged cystic lesion in the left frontal lobe (figure not shown).

The diagnosis of parasitosis was confirmed at follow-up, and the patient was prescribed one course of praziquantel, which was ineffective. The headache worsened, and after 3 months, the patient was admitted to our hospital.

**Figure 4:**
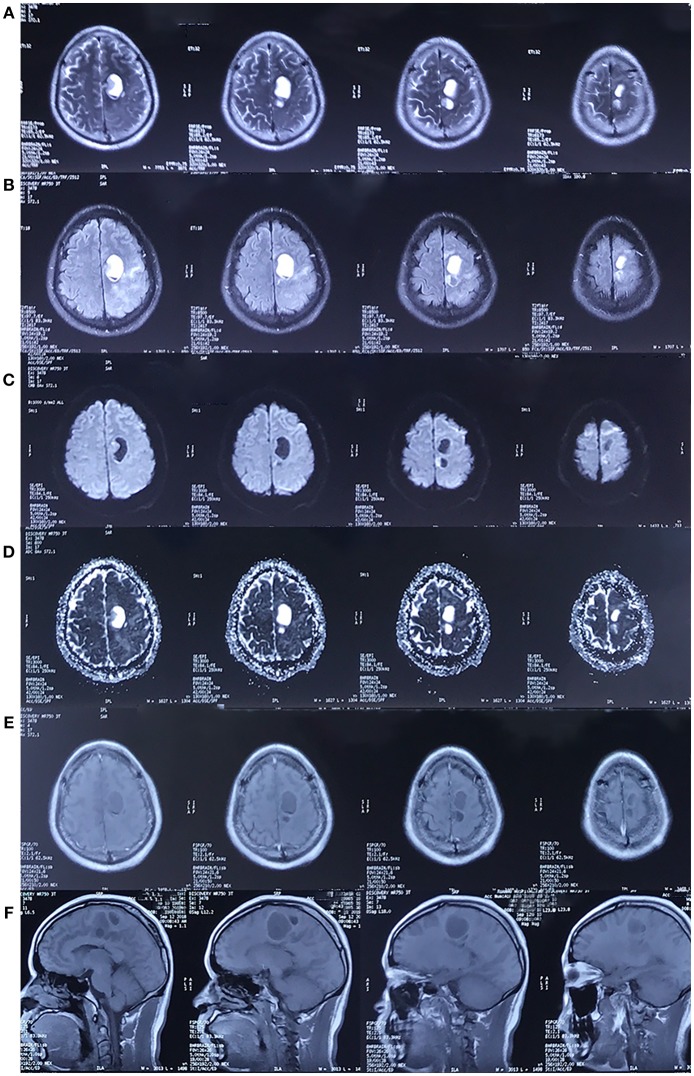
MRI in September 2018. The cystic lesion in left frontal lobe was enlarged. The lesion showed the same characteristics as the MRI scan performed in 2017. [**(A–F)** Represents T2-weighted image, FLAIR, DWI, ADC, axial and sagittal post-contrast T1-weighted image, respectively. The pre-contrast T1-weighted image was unavailable].

**Figure 5:**
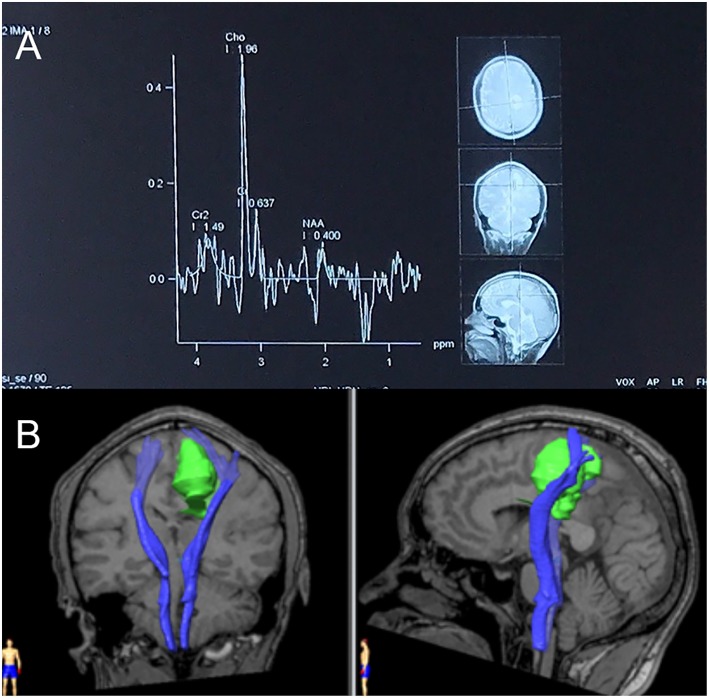
**(A)** Magnetic resonance spectroscopy (MRS) in September 2018. MRS of peri-enhancement area detected a significantly heightened choline (Cho) peak at 3.2 ppm, and a weakened N-acetylaspartate (NAA) peak at 2.0 ppm. Resonance peak integral of Cho and NAA were 1.96 and 0.40, respectively. The Cho/NAA ratio was 4.9. **(B)** Diffusion tensor imaging (DTI) in September 2018. Three-dimensional reconstruction images showed an invasion of the lesion into the left pyramidal tract.

Upon admission, a physical examination showed weakness of the right leg, which was worse in the distal (0/5) than in the proximal (4/5) muscles, with an ipsilateral hyperactive knee reflex and positive Chaddock's sign. An MRI scan revealed further enlargement of the lesion (Figure 4), and magnetic resonance spectroscopy (MRS, Figure 5A) and diffusion tensor imaging (DTI, Figure 5B) were scheduled. MRS detected a significantly heightened choline (Cho) peak and a weakened N-acetylaspartate (NAA) peak. DTI showed an invasion into the left pyramidal tract.

**Figure 6:**
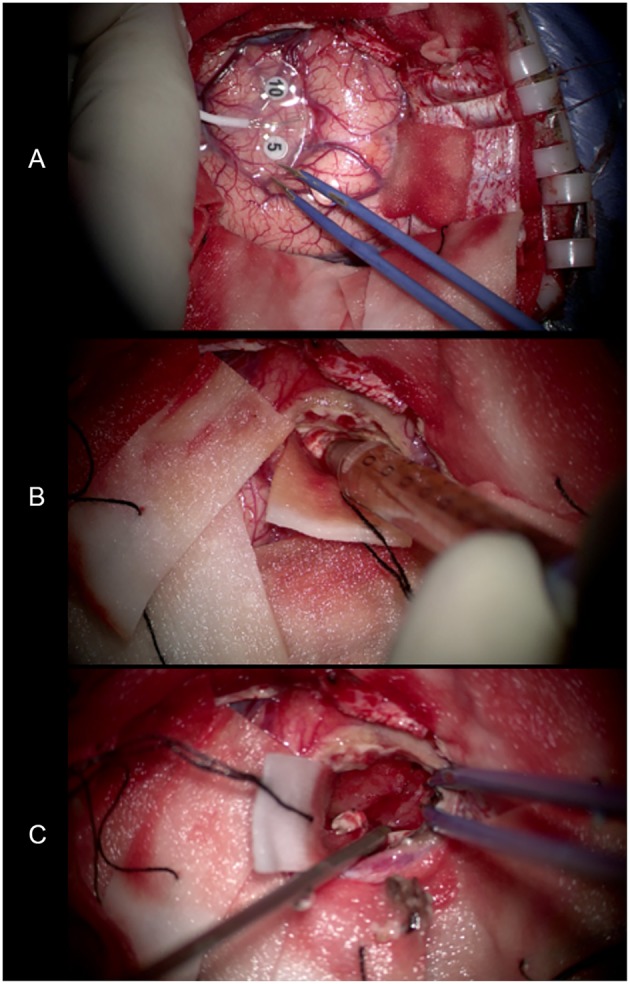
**(A)** Use of an intraoperative electrophysiological monitor during surgery. Electrodes were placed on the surface of the brain to identify the functional area. **(B)** The pale-yellow cystic fluid suctioned by the needle. **(C)** The cyst wall exposed after removing the cystic fluid. It was soft and dark red in appearance.

We performed lesion resection under an intraoperative electrophysiological monitor to protect the adjoining precentral gyrus (Figure 6A). Swelling of the left frontal cortex, which was close to the midline, came into view as soon as the brain was exposed. Beneath the cortex, there was a solid-cystic lesion containing pale-yellow cystic fluid (Figure 6B). No scolex or larvae were seen. The cyst wall was soft and dark red in appearance (Figure 6C). The intraoperative report indicated high-grade glioma.

**Figure 7:**
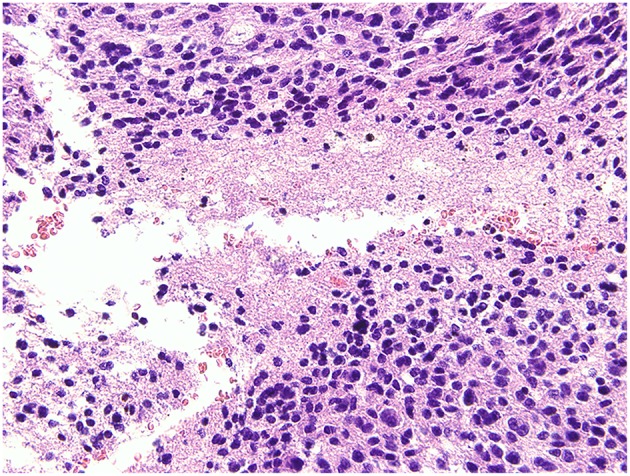
Histological analysis. The pathological report gave a diagnosis of glioblastoma, WHO IV, without mutation of the isocitrate dehydrogenase 1 (IDH1) gene.

The postoperative pathological findings indicated glioma, classified as glioblastoma, WHO IV according to 2016 World Health Organization (WHO) Classification of Tumors of the Central Nervous System. Histological analysis showed an absence of mutation of the isocitrate dehydrogenase 1 (IDH1) gene (Figure 7).

Written consent for publication of this case report was obtained from the patient.

## Discussion

We describe a case of a patient with secondary glioblastoma who was mistakenly diagnosed with parasitosis and received inappropriate treatment. In view of the slow progression of the disease, an early diagnosis would have resulted in a better prognosis. There were several reasons for the incorrect diagnosis: inadequate attention to the initial abnormality on the MRI scan; insufficient knowledge on the clinical features of different parasitosis; and being misled by the patient's raw food diet, serological antigen positivity and FDG-PET results.

### Initial MRI Results

The first MRI scan was conducted in 2013, ~2 years after the onset of disease. This scan showed an abnormal signal in the left frontal cortex that was barely discerned in the absence of enhancement.

Conventional MRI, which includes T2-weighted and pre- and post-gadolinium contrast-enhanced T1-weighted images, can provide sufficient anatomic details. It is often used in the diagnosis of brain tumors (3, 4). In the absence of cellular, metabolic and vascular information, however, it is difficult to differentiate neoplastic brain masses from non-neoplastic brain masses. Thus, physiology-based MRI methods, such as diffusion-weighted imaging (DWI), MRS, and perfusion-weighted imaging (PWI), are often used to increase the sensitivity and specificity of the detection (5, 6) and the grading (7) of brain tumors. Although the patient underwent DWI (Figure 2) in 2017, MRS (Figure 5A) in 2018, and DTI (Figure 5B) in 2018, all of which indicated glioblastoma, the opportunity for a timely and appropriate treatment has gone.

Al-Okaili et al. histologically confirmed the effectiveness of a multimode MRI-based approach (8) in differentiating intra-axial brain masses. Its accuracy, sensitivity, and specificity were 90, 97, and 67%, respectively, for the discrimination of neoplastic lesions from non-neoplastic lesions, and 90, 88, and 100%, respectively, for the discrimination of high-grade neoplasms from low-grade neoplasms. According to this strategy, MRS follows MRI to further differentiate an unenhanced intracerebral lesion. Low grade neoplasm or encephalitis are considered when there is elevation of Cho/NAA ratio over 2.2 in an unenhanced brain lesion. Even with the ratio <2.2, low-grade neoplasms cannot be excluded and further follow-up or biopsy is required.

In our case, the patient underwent only conventional MRI and was followed-up for the abnormal signal. Although this decision seemed reasonable at the time, we should have handled the case with higher vigilance and requested more frequent follow-ups, instead of a 4-year gap without re-examination.

### Follow-Up MRI Results

The second MRI scan was conducted in 2017. The scan revealed a cystic lesion at the same region, which presented as a low signal on T1-weighted image, a high signal on T2/T2-FLAIR with surrounding edema, and a low signal on DWI. Post-contrast T1-weighted image revealed rim enhancement. A third MRI scan was conducted in 2018, and the image was identical to the scan from 2017, except that the lesion was further enlarged.

In general, gliomas are solid tumors with or without enhancement (9). The formation of cyst raises the possibility of a high-grade glial neoplasm (10). In the MRI scan in 2017, different from the typical enhancement features of glioblastoma, where there tend to be an irregular, garland-shaped rim (11), the cyst was oval and had relatively smooth inner margin. Thus, it was challenging to differentiate it from benign lesions with similar manifestation. Cysticercosis, abscesses, and tuberculomas (12, 13) are among the most common ones.

Nevertheless, the DWI provided us with critical information. Previous studies (14–17) have revealed that DWI can differentiate cystic intracerebral lesions. Both high-grade gliomas and metastases are hypointense on DWI scans and can reveal high diffusion on Apparent diffusion coefficient (ADC) maps (15) as a result of facilitated diffusion. On the contrary, abscesses (16) usually appear with limited diffusion and are hyperintense on DWI scans. With regard to the MRI-based approach (8), an enhanced lesion with facilitated diffusion should have been diagnosed as a high-grade neoplasm.

At the time of diagnosis, however, the patient's special diet, relative atypical enhancement pattern and PET-CT results were misleading, and the MRI results were mistaken for cystic parasitosis with central necrosis. It must be noted that liquefactive necrotic area can also exhibit hypointense lesions on DWI with increased ADC, reflecting the process of encephalomalacia with unrestricted water motion (18), but rim enhancement is hardly the feature of *A. cantonensis* infection, as discussed blow.

MRS revealed a heightened Cho peak and a weakened NAA peak, with a Cho/NAA ratio of 4.9. The level of Cho/NAA ratio helps distinguish different grades of glioma (19). It is significantly higher in HGGs (3.86 ± 3.31) and lower in LGGs (0.81 ± 0.90) (20). Following an established diagnosis of high-grade neoplasm based on MRI, a Cho/NAA ratio over 1 in peri-enhancement area leads to the diagnosis of high-grade glioma (8).

A DTI scan, which helped us to understand the relationship between the tumor and the ipsilateral pyramidal tract, was performed 9 days before the surgery. DTI is based on diffusion measurements, and its parameters (i.e., apparent diffusion coefficient [ADC], mean diffusivity [MD], and fractional anisotropy [FA]) reflect the integrity of the white matter and tumor infiltration (21, 22). Due to differences in these characteristics, both LGGs and high grade gliomas (HGGs) (23), as well as HGGs and brain metastases (24), can be discriminated. An arithmetic formula combining multiple parameters can achieve a sensitivity of 91.7% and a specificity of 86.4% when differentiating LGGs from metastases (25), as well as a sensitivity of 84.8%, a specificity of 74.5%, and an accuracy of 80.4% when differentiating HGGs from LGGs (26).

**Figure 8:**
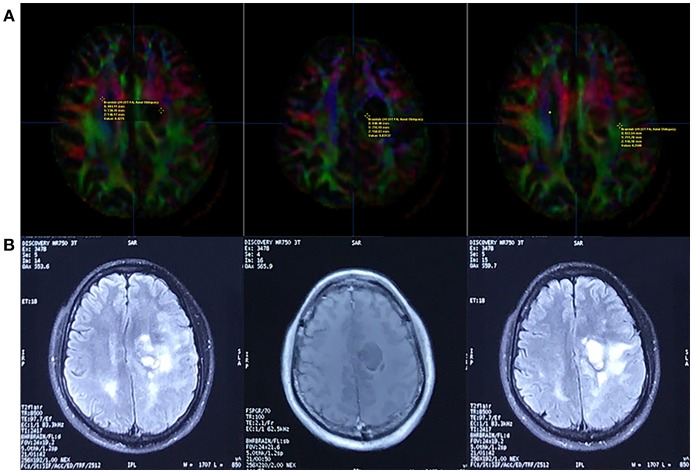
Fractional Anisotropy (FA) parametric map based on DTI **(A)** and corresponding FLAIR/contrast-enhanced T1-weighted volume images **(B)**. The FA value of the contralateral normal white matter, non-enhancing tumor core, and edematous brain was 0.4275, 0.03137, and 0.2588, respectively (line A, from left to right).

We measured the FA value in the non-enhancing tumor core, edematous brain, and contralateral normal white matter (Figure 8) and found that the distribution was consistent with that of earlier studies on HGGs. The non-enhancing tumor core had the lowest FA value, and the normal white matter had the highest (27).

### FDG-PET Results

A PET-CT scan was conducted in October 2017, <1 month after the second MRI scan. The report revealed “decreased glucose metabolism, consistent with a primary benign lesion.” Combined with the patient's history of eating raw meat and the positive serological findings, clinicians arrived at an incorrect diagnosis, which affected the treatment protocol.

Low-grade gliomas tend to exhibit low uptake of glucose, whereas high-grade gliomas tend to exhibit high uptake of FDG compared with normal gray and white matter (28, 29). Based on the MRI scan from 2017, we considered a high-grade tumor and deemed the PET-CT results as unreasonable.

Studies have reported that FDG-PET can reveal hypo- or iso-metabolism in high-grade gliomas (30, 31). The fact that normal brain tissue has the highest glucose metabolic rate in the body implies the intrinsic shortage of glucose-metabolism-based FDG-PET in the detection of intracerebral tumors.

New non-FDG tracers for PET-CT are available. Amino acid based tracers, such as [^11^C]methionine ([^11^C]MET) (32, 33) and [^18^F]fluoroethyl-L-tyrosine ([^18^F]FET) (34), are ideal imaging biomarkers of tumor growth, since they are rarely up-taken by cells in the normal brain but accumulate in proliferating cells as a result of up-regulated protein synthesis.

According to a previous study, FET sensitivity was 93%, specificity 100%, accuracy 96%, positive predictive value (PPV) 100% and negative predictive value (NPV) 91% for detecting malignant brain tumors, while FDG sensitivity was 27%, specificity 90%, accuracy 52%, PPV 80%, and NPV 45%(35). Other studies (36, 37) also revealed a significantly better performance of FET compared to FDG-PET in the detection of brain tumors. In addition, a previous study revealed that brain lesions showing hypo- or iso-metabolism on FDG-PET can be detected and differentiated with high sensitivity using MET-PET (31). Taking all these into consideration, we would have arrived at a different diagnosis if amino acid-based PET was used instead of, or in addition to, FDG-PET alone.

### Personal History and Serological Results

The patient's history of eating raw meat led the attending physician to screen for a parasitic infection.

Most cases of cerebral parasitosis derive from cerebral cysticercosis, whose imaging features are variable (38, 39). Solitary parenchymal cyst (SCC) (40, 41) is one of its most common manifestations, with an incidence of approximately 23.59% (42). Degenerating cysts can appear as single contrast-enhancing rings surrounded by edema, which could be confused with gliomas (43, 44).

However, it is important to point out that the serological results indicated the presence of *A. cantonensis* IgG instead of cysticercosis. *A. cantonensis* infects humans through third-stage larvae from snails, slugs, or contaminated, uncooked vegetables (45, 46). The typical manifestation of *A. cantonensis* infection is eosinophilic meningitis (47, 48). It can present as a disc lesion after the formation of an abscess (49), but a brain abscess exhibits distinct features in DWI, as we discussed above. In this case, there was no evidence of meningitis, no increase in serum or CSF eosinophils, and the PCR results were negative for *A. cantonensis* infection, which was inconsistent with the serological results. PCR, as a molecular approach that can detect a single molecule of DNA, has a better sensitivity in detecting parasites than morphological and biological techniques (50, 51). Thus, it was very likely that the patient was not infected with *A. cantonensis*.

The pathological findings confirmed the diagnosis of glioblastoma, which is classified as WHO IV according to 2016 World Health Organization (WHO) Classification of Tumors of the Central Nervous System (52). Based on clinical manifestations, this case was typical of secondary glioblastoma, with neuroimaging evidence of an evolution from a less malignant astrocytoma (53). According to a previous population-based study (1), this type of glioma is very rare, accounting for only 5.3% of all glioblastomas. By contrast, primary or *de-novo* glioblastomas, which manifest rapidly (clinical history is <3 months in the majority of cases) without radiological or histological evidence of a less malignant precursor lesion, account for 94.7% of all glioblastomas. Our patient was 46 years old at disease onset, which is close to the mean onset age (i.e., 45 years) of secondary glioblastoma, whereas primary glioblastomas develop in older patients, with a mean age of 62 years (1).

What is remarkable is that, despite a clinical slow-growing feature, the tumor turned out to be IDH-wildtype, which is a minority in secondary glioblastomas and might imply a distinct histological origin (54) from the majority.

The patient underwent normative resection and complementary treatment immediately after confirmation of the pathology. We also arrived at several important considerations after all these twists and turns.

Firstly, early stage malignant gliomas can manifest neither specific clinical signs such as epileptic seizure or focal neurological deficits, nor imaging signs of necrosis or significant enhancement (55). A comprehensive imaging protocol, one that involves multi-parametric MRI, is necessary and efficient when dealing with undefined cerebral lesions.

Secondly, a low glucose metabolism is not always indicative of a benign lesion. It is critical for clinicians to recognize the limitations of FDG-PET in cerebral imaging.

Thirdly, when trying to make a diagnosis of a rare disease, such as brain *A. cantonensis* infection, it is important to fully understanding its manifestations and carefully gather clues.

Last but not least, consecutive follow-ups are indispensable in such cases. A comparative research in 2012 proved that early resection of LGGs was associated with better overall survival compared to the “biopsy and watchful waiting” strategy (56). Supposing that the patient in our case had more frequent follow-up and early biopsy, an early diagnosis and resection could be possible. As malignant neoplasm can have subtle imaging appearances in early stage, clinicians should always be vigilant.

## Data Availability

All datasets generated for this study are included in the manuscript/supplementary files.

## Author Contributions

CL: collected and analyzed patient information and wrote the manuscript. WX: performed the surgery. PL: analyzed and rebuilt DTI. YW: performed the surgery and reviewed the manuscript.

### Conflict of Interest Statement

The authors declare that the research was conducted in the absence of any commercial or financial relationships that could be construed as a potential conflict of interest.
